# Safe and effective antireflux surgery in lung transplant recipients: preliminary results

**DOI:** 10.20452/wiitm.2025.17963

**Published:** 2025-07-04

**Authors:** Maciej Wiewiora, Marek Ochman, Maciej Urlik, Katarzyna Hajduk, Aleksandra Gil, Konrad Karcz, Tomasz Hrapkowicz

**Affiliations:** Department of General Surgery, Vascular Surgery, Angiology and Phlebology, Faculty of Medical Sciences in Katowice, Medical University of Silesia, Katowice, Poland; Department of Cardiac Vascular and Endovascular Surgery and Transplantology, Faculty of Medical Sciences in Zabrze, Medical University of Silesia in Katowice, Zabrze, Poland; Student Scientific Society, Department of Cardiac, Vascular and Endovascular Surgery and Transplantology, Faculty of Medical Sciences in Zabrze, Medical University of Silesia in Katowice, Zabrze, Poland; Department of General, Visceral and Transplant Surgery, University Hospital, Ludwig Maximilian University in Munich, Munich, Poland

**Keywords:** antireflux surgery, bronchiolitis, lung transplantation, obliterans syndrome

## Abstract

**INTRODUCTION:**

Obliterative bronchiolitis, the clinical manifestation of bronchiolitis obliterans syndrome (BOS), is a major complication of lung transplantation and one of the primary causes of chronic lung allograft dysfunction leading to poor survival outcomes.

**AIM:**

The aim of this study was to evaluate the safety and outcomes of antireflux surgery in lung transplant recipients (LTRs) with BOS and associated gastroesophageal reflux disease (GERD).

**MATERIALS AND METHODS:**

This single-center study included 8 consecutive patients at a median (interquartile range [IQR]) age of 29 (25–46) years who underwent bilateral lung transplantation and subsequent antireflux surgery due to BOS. The decision to proceed with laparoscopic surgery was based on a diagnosis of GERD associated with a decline in pulmonary function, confirmed on bronchoscopy indicative of BOS. Follow-up lasted for 12 months.

**RESULTS:**

Median (IQR) time since transplantation was 27 (15–55.5) months. Significant improvements in spirometric parameters were observed at 3 and 12 months postoperatively, as compared with baseline, including forced expiratory volume in 1 second (FEV_1_; *P* = 0.02), FEV_1_% predicted (*P* = 0.02), forced vital capacity (FVC; *P* = 0.003), and FVC% predicted (*P* = 0.02). There were no differences in spirometric parameters between 3 and 12 months postoperatively. No surgical complications were observed within 30 days after surgery or during follow-up. Two patients developed pulmonary complications, and 1 patient with a history of kidney transplantation experienced renal complications. There were no postoperative deaths. Median (IQR) hospital stay was 12 (3–29) days.

**CONCLUSIONS:**

The study suggests that antireflux surgery in LTRs is a safe and effective approach for GERD management, while improving lung function with minimal adverse effects.

## INTRODUCTION

Lung transplantation is a treatment modality that improves quality of life and extends survival for patients with end-stage lung diseases. Although long-term survival rates have improved over the years, the 5-year graft survival rate is approximately 50%, which is lower than in the case of other solid organ transplants.^1^ Obliterative bronchiolitis, the clinical manifestation of bronchiolitis obliterans syndrome (BOS), is a major complication of lung transplantation and one of the primary causes of chronic lung allograft dysfunction (CLAD) leading to poor survival outcomes. BOS is defined as an obstructive ventilatory defect with a persistent decline in forced expiratory volume in 1 second (FEV_1_), as compared with the post-transplant baseline, in the absence of other identifiable causes.^2^

It is estimated that 40%–50% of lung transplant recipients (LTRs) develop BOS within 5 years postoperatively, with a median (interquartile range [IQR]) survival of 3.5 (3–5) years following its onset.^3^ CLAD is associated with a significant decline in lung function after lung transplantation, in the absence of other identifiable causes. It is defined as a persistent decline (≥20%) in FEV_1_, as compared with the post-transplant baseline, which is itself defined as the average of the 2 maximal post-transplant FEV_1_ values that are at least 3 weeks apart. The International Society for Heart and Lung Transplantation guidelines^4^ indicate 2 main phenotypes of CLAD that should be identified at the time of CLAD onset, based on the measurements of FEV_1_ and forced vital capacity (FVC): BOS and restrictive allograft syndrome. The key symptom of BOS is airflow limitation, so spirometry is a standard method for monitoring LTRs.^4^

**TABLE 1:** Baseline characteristics of the study patients

Parameter	Value
Age, y	29 (25–46.5)
Male sex	6 (75)
Weight, kg	63 (53.5–78)
BMI, kg/m²	21.5 (18.5–25)
SBP, mm Hg	125 (120–135)
DBP, mm Hg	80 (72.5–85)
Time from lung transplantation, mo	27.5 (15–55.5)
Intraoperative time, min	140 (125–180)
Blood loss, ml	100 (60–140)
Length of hospital stay, d	5 (4–12)
Comorbidities	
Hiatal hernia	8 (100)
GERD	8 (100)
Diabetes mellitus	4 (50)
Arterial hypertension	4 (50)
Cystic fibrosis	3 (37.5)
Chronic pancreatitis	3 (37.5)
Gout	4 (50)
Chronic kidney disease	2 (25)
Heart failure	1 (12.5)
Primary pulmonary fibrosis	1 (12.5)
Pulmonary hypertension	1 (12.5)

Data are presented as median (interquartile range) or number (percentage).

Abbreviations: BMI, body mass index; DBP, diastolic blood pressure;

GERD, gastroesophageal reflux disease; SBP, systolic blood pressure

**TABLE 2:** Early postoperative complications

Complication	Number, %
Bleeding	–
Leakage	–
Reoperation	–
Other surgical complications	–
Heart failure	–
Respiratory failure	2 (25)
Major arrhythmia	–
Hypotension	–
Acute kidney injury	1 (12.5)
Pneumonia/sepsis	1 (12.5)
Myocardial ischemia/infarction	–
Stroke	–

Gastroesophageal reflux disease (GERD) has been implicated as a nonimmune contributor to BOS development and a potential risk factor for allograft injury.^5^ Although the exact mechanism through which GERD contributes to BOS remains unclear, numerous studies have demonstrated a correlation between GERD-related aspiration and the onset of lung diseases, including BOS.^6^ Antireflux surgery has become a standard treatment for GERD in LTRs, although a direct causal relationship between GERD and BOS is yet to be definitively established.

## AIM

The primary aim of this study was to evaluate the safety and outcomes of antireflux surgery in LTRs with BOS and associated GERD. The secondary end point was to evaluate the improvement of lung function after antireflux surgery.

## MATERIALS AND METHODS

### Study characteristics

This observational study focused on patients who underwent bilateral lung transplantation followed by antireflux surgery due to BOS associated with GERD and / or hiatal hernia in 2018, 2023, and 2024. The study included 8 consecutive patients after antireflux surgery with 12-month follow-up.

Consecutive post-transplant patients with deteriorating lung function were qualified for surgery if they met generally accepted criteria for an antireflux procedure, namely: symptoms of BOS, deteriorating lung function as indicated by a decrease in FEV_1_, and endoscopically confirmed GERD. CLAD staging was performed to stratify potential investigations and therapies.^7^ Patients with BOS due to other causes or without confirmed GERD were treated conservatively. The surgeries were performed at a single center by transplant surgeons and general surgeons with extensive experience in laparoscopy. The decision to operate was made by a team consisting of a transplantologist, a pulmonologist, and a surgeon. All patients had GERD confirmed on endoscopy and underwent manometry. The first-choice procedure was Toupet fundoplication. In the cases where no esophageal motility disorders were found, the patient was qualified for a Nissen fundoplication.

Baseline characteristics, including age, sex, body mass, body mass index, and comorbidities, were recorded. The primary outcome was the occurrence of early and late complications resulting from the surgical procedure. Major complications in the early postoperative period included the need for transfusion of at least 2 units of packed red blood cells, bleeding requiring reoperation, complications necessitating reoperation or readmission, or death. Early complications were defined as those occurring within 30 days postoperatively, while late complications were understood as those occurring beyond 30 days after surgery and requiring a secondary procedure or readmission. Surgical complications were assessed during hospitalization and throughout follow-up. Secondary outcomes included changes in spirometric parameters during follow-up. The decision to proceed with laparoscopic surgery was based on a GERD diagnosis associated with a decline in pulmonary function, confirmed on bronchoscopy indicative of BOS. All patients had persistent GERD symptoms before antireflux surgery despite receiving regular proton pump inhibitor (PPI) therapy. Six patients underwent laparoscopic Toupet fundoplication, while 2 underwent laparoscopic Nissen fundoplication. The patients were followed by the surgical team for 12 months.

**FIGURE 1:**
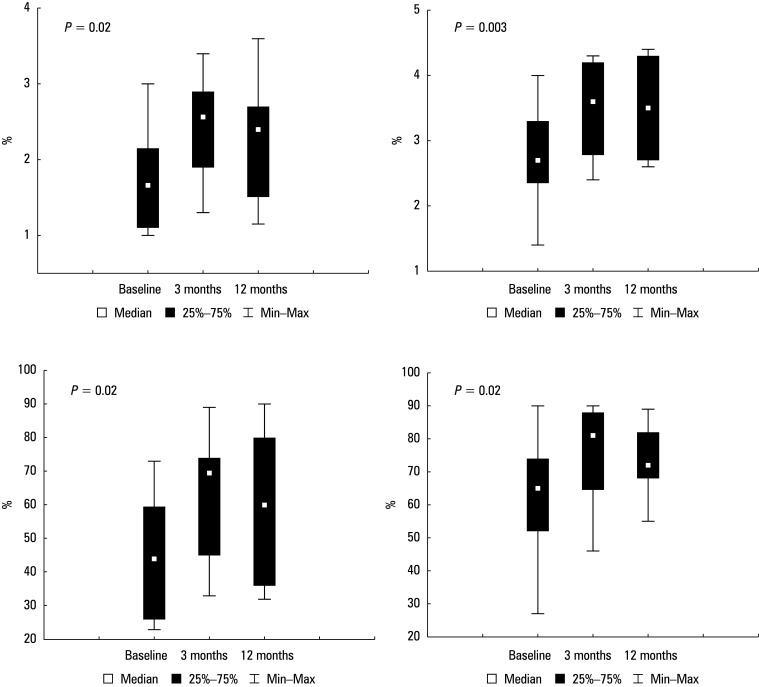
Changes in spirometric parameters observed at 3 and 12 months after antireflux surgery, as compared with baseline; A – FEV1; B – FEV1% predicted; C – FVC; D – FVC% predicted

Abbreviations: FEV, forced expiratory volume; FVC, forced vital capacity

### Esophageal assessment

Each patient was diagnosed with GERD based on evidence of pathological esophageal acid exposure and persistent symptoms. All patients were treated with PPIs after lung transplantation, but these failed to alleviate reflux symptoms. Endoscopic findings included erosions and hiatal hernia. No complications related to the endoscopic procedure were observed.

### Pulmonary assessment

The patients underwent pulmonary function tests, including spirometry, which assessed FVC and FEV_1_. FEV_1_% predicted, defined as FEV_1_% of the patient divided by the average FEV_1_% in the population for any person of similar age, sex, and body composition, was also calculated. Surveillance bronchoscopy was performed in all patients, and the presence of vegetable material in biopsy specimens was considered indicative of gastroesophageal reflux-related aspiration.

### Surgical technique

Two types of antireflux surgery were performed: laparoscopic partial Toupet fundoplication and complete Nissen fundoplication. Both procedures were performed under general anesthesia using 4 trocars and included hiatal hernia repair. The first step involved exploring the hiatal hernia to assess its size and the mobility of the distal esophagus. Next, the right hiatal crus, the hiatal arch, and the left crus were identified to establish the correct dissection plane and create the so-called “gastroesophageal window” posterior to the esophageal wall. The gastric fundus and greater curvature were mobilized by dividing the short gastric vessels. The hiatus was partially closed posteriorly by approximating the left and right crura, using nonabsorbable sutures, calibrated with a 45 French bougie to maintain the minimum diameter. Once the bougie was in place within the gastric lumen, the posterior and anterior fundic flaps were wrapped around the distal esophagus. For the Nissen fundoplication, the posterior and anterior flaps were secured using 3 nonabsorbable sutures to create a short, floppy wrap. For the Toupet procedure, the fundic flap was passed through the gastroesophageal window from left to right and secured to the right diaphragmatic crus with 3 sutures. Additionally, the fundus was sutured to the distal esophagus. All surgeries were performed by 2 experienced surgeons.

### Statistical analysis

The Shapiro–Wilk test was used to test normal distribution in all continuous variables. Continuous variables were presented as medians (IQR) if they were not normally distributed. Categorical variables were presented as absolute numbers and percentages. Differences in spirometric parameters before surgery and at 3 and 12 months postoperatively were analyzed using the Friedman analysis of variance test. Statistical analyses were performed using Statistica 12 package (StatSoft, Inc., Tulsa, Oklahoma, United States). A *P* value below 0.05 was considered significant.

## RESULTS

Baseline characteristics of the study group are presented in Table 1. The study included patients who were followed for 12 months with complete pre- and postoperative data. The cohort consisted of 6 men and 2 women at a median (IQR) age of 29 (25–46) years. Median (IQR) time since transplantation was 27 (15–55.5) months. The most common comorbidity was diabetes mellitus, diagnosed in 5 patients. Four of them required insulin therapy, while 1 was managed with metformin. Additional comorbidities included arterial hypertension (4 patients), gout (4 patients), cystic fibrosis with chronic pancreatitis (3 patients), and chronic kidney disease (2 patients). One patient had primary pulmonary fibrosis with associated pulmonary hypertension. Among them, 6 underwent laparoscopic Toupet fundoplication, and 2, laparoscopic Nissen fundoplication.

Before surgery, FVC values were reduced, indicating varying degrees of impaired lung capacity. Spirometric analysis showed a reduction in FEV_1_ in all patients prior to surgery. Two patients had CLAD stage 2, 4 participants had CLAD stage 3, and 2, CLAD stage 4. Significant improvements in spirometric parameters were observed at 3 and 12 months postoperatively in comparison with baseline (Figure 1A**–**1D). Median (IQR) FEV_1_ and FEV_1_% predicted at baseline, 3 months, and 12 months after surgery were 1.66 l, 2.56 l, and 2.4 l , respectively (P = 0.02), and 44%, 69.5%, and 60%, respectively (*P* = 0.02; Figure 1A and 1B). Median (IQR) FVC and FVC% predicted at the same times were 2.7 l, 3.6 l, and 3.5 l (*P* = 0.003), respectively, and 65%, 81%, and 72%, respectively (*P* = 0.02; Figure 1C and 1D). There were no differences in spirometric parameters between 3 and 12 months after surgery. Median FVC and FEV_1_ increased considerably at these times and remained stable throughout follow-up. No intraoperative complications or postoperative hemorrhages were observed (Table 2). There were no surgical complications within 30 days after the procedure or during follow-up. Two patients who had undergone Toupet fundoplication developed respiratory failure related to pre-existing cystic fibrosis. One patient with a history of kidney transplantation, also after Toupet fundoplication, experienced renal complications. One participant after Nissen fundoplication had pneumonia. There were no postoperative deaths. Median (IQR) hospital stay was 12 (3–29) days.

## DISCUSSION

A significant contributor to post-transplant mortality in LTRs is BOS, characterized by a persistent reduction in FEV_1_, which over time leads to CLAD.^8^ The pathogenesis of CLAD remains poorly understood, with multiple factors implicated in BOS development. GERD commonly affects individuals awaiting lung transplantation due to end-stage lung disease, as well as those who have already undergone the procedure.^9^ Worsening of GERD post-transplantation has been well documented.^10^

The presence of gastroesophageal reflux in LTRs raises concerns for 2 primary reasons. First, it increases the risk of aspiration-related allograft injury, potentially contributing to allograft rejection. Second, there is associated morbidity linked with post-transplantation fundoplication which currently is the only effective treatment for preventing reflux-related aspiration in this specific patient population.^11^**^,^**^12^ GERD is considered a significant comorbidity in LTRs and may be linked to the development of obliterative bronchiolitis.^13^ Recent studies suggest that antireflux surgery may offer protection against CLAD by reversing the decline in FEV_1 _following transplantation.^14^

Fundoplication is a surgical procedure commonly used to treat GERD. However, its application in LTRs remains relatively uncommon, primarily due to the prevalence of noninvasive treatments, such as PPIs, and concerns regarding whether the theoretical benefits of early antireflux surgery outweigh the risks of a surgical procedure in high-risk, immunocompromised patients.^15^ The risks and technical limitations of antireflux surgery in this population have been described, including an increased risk of gastroparesis and tissue friability due to steroid use. However, studies from various groups suggest^16^**^,^**^17^ that fundoplication represents a safe and effective strategy for GERD management in LTRs.

The present study indicates that laparoscopic fundoplication in LTRs is a safe procedure. There were no surgical complications or reoperations in the early postoperative period or during follow-up. No cases of bleeding or leakage were observed. In the early postoperative period, 3 patients experienced complications related to pulmonary dysfunction. Two participants with cystic fibrosis had postoperative pulmonary dysfunction requiring prolonged stay in the intensive care unit with mechanical ventilation followed by oxygen therapy. One patient developed pneumonia without accompanying respiratory failure, which required standard treatment. Postoperative renal dysfunction occurred in 1 participant with a history of kidney transplantation. This may have been related to inadequate perioperative fluid management. Our analysis showed a significant increase in spirometric parameters in the studied group, suggesting that antireflux surgery may play a protective role in preventing lung function deterioration in individuals with GERD. Fundoplication was a safe procedure in our transplant recipients, its safety profile comparable to that in patients without a history of lung transplantation. However, the duration of surgery in our patient group was longer than that observed in the general population.^18^

A recent multicenter retrospective study^19^ found that, following transplantation, the patients who underwent fundoplication had a lower incidence of primary graft dysfunction (2.2%), as compared with the GERD group (17.7%), and shorter median (IQR) hospital stay. The fundoplication group also recorded a slightly better mortality rate and increased freedom from obliterative bronchiolitis. Additionally, the patients who underwent fundoplication had higher observed FEV_1_ values up to 5 years post-transplantation. Similar results were obtained by other researchers who found that fundoplication after lung transplantation reduced the rate of FEV_1_ decline.^20^

Yergin et al^21^ conducted a prospective case series of LTRs who underwent laparoscopic Toupet fundoplication. They found that the procedure provided objective control of pathological acid reflux and symptomatic improvement in gastroesophageal reflux while preserving lung function. However, there were no significant differences in the overall rate of FEV_1_ change after fundoplication. Notably, the patients who experienced a decreasing rate of FEV_1_ prior to fundoplication saw an improvement in their rate of change post-fundoplication. This is consistent with our findings, as all patients in our study group who underwent fundoplication had declining pulmonary function preoperatively. We observed improvements in spirometric parameters at 3 months after surgery, which persisted throughout 12-month follow-up. A recent meta-analysis^14^ exploring lung function in LTRs before and after antireflux surgery found that both FEV_1_ and the pooled rate of change in FEV_1_ improved, concluding that fundoplication was beneficial and potentially protective against CLAD.

For surgery, we selected patients who were diagnosed with BOS associated with GERD and hiatal hernia. In our patient group, median (IQR) time from lung transplantation to surgery exceeded 24 (15–55) months, as we included individuals with diagnosed GERD and confirmed BOS. Biswas et al^9^ advocate for early antireflux surgery to mitigate the risk of disease progression. They found that patients who underwent early fundoplication had higher FEV_1_ values 5 years after lung transplantation, suggesting that this intervention may protect against GERD-induced lung damage.

A recent study^22^ indicates that LTRs with GERD who underwent antireflux surgery had superior pulmonary function and longer CLAD-free survival than those managed medically. This was particularly evident in patients who underwent early fundoplication rather than late intervention.^22^ Another recent study^23^ reaffirmed that LTRs do not face greater surgical risks than the general population undergoing laparoscopic fundoplication. On the contrary, this intervention is likely to improve their survival rate. A recent systematic review and meta-analysis^24^ confirmed that antireflux surgery improved FEV_1_, potentially preventing CLAD and improving allograft survival. The authors also emphasized that, given the increased incidence of GERD in LTRs, there should be a low threshold for investigating GERD and considering antireflux surgery.

### Study limitations

This study has several limitations. First, the patient population was small, which was mainly due to the specific nature of the patient group, as they were rarely qualified for surgery. The observational, nonprospective nature of the study introduces a risk of missing data regarding long-term follow-up. In our study, follow-up was 12 months with complete clinical and spirometric data. Another limitation is the inability to compare the 2 fundoplication methods in this particular group of patients. Finally, the number of procedures performed was significantly affected by the COVID-19 pandemic, during which this type of surgery was not performed at all.

## CONCLUSIONS

This study suggests that antireflux surgery in LTRs is a safe and effective surgical approach for managing GERD while improving lung function with minimal adverse effects. However, due to the limited sample size and short follow-up, general recommendations for antireflux surgery in this patient population cannot yet be made. Further prospective, randomized studies are warranted to confirm these findings.

## JOURNAL INFORMATION

Videosurgery and Other Miniinvasive Techniques is an official journal of the Videosurgery Foundation.

## References

[BIBR-1] Valapour M., Lehr C.J., Skeans M.A. (2018). OPTN/SRTR 2018 annual data report: lung. Am J Transplant.

[BIBR-2] Meyer K.C., Raghu G., Verleden G.M. (2014). An international ISHLT/ATS/ERS clinical practice guideline: diagnosis and management of bronchiolitis oblit‐ erans syndrome. Eur Respir J.

[BIBR-3] Kemp R., Pustulka I., Boerner G. (2021). Relationship between FEV1 decline and mortality in patients with bronchiolitis obliterans syndrome – a system‐ atic literature review. Respir Med.

[BIBR-4] Estenne M., Maurer Boehler (2002). Bronchiolitis obliterans syn‐ drome 2001: an update of the diagnostic criteria. J Heart Lung Transplant.

[BIBR-5] Hathorn K.E., Chan W.W., Lo W.K. (2017). Role of gastroesophageal reflux disease in lung transplantation. World J Transplant.

[BIBR-6] Patti M.G., Vela M.F., Odell D.D. (2016). The intersection of GERD, aspira‐ tion, and lung transplantation. J Laparoendosc Adv Surg Tech A.

[BIBR-7] Verleden G.M., Glanville A.R., Lease E.D. (2019). Chronic lung allograft dys‐ function: definition, diagnostic criteria, and approaches to treatment: a con‐ sensus report from the Pulmonary Council of the ISHLT. J Heart Lung Trans‐ plant.

[BIBR-8] Verleden G.M., Raghu G., Meyer K.C. (2014). A new classification system for chronic lung allograft dysfunction. J Heart Lung Transplant.

[BIBR-9] S Biswas Roy, S Elnahas, R Serrone (2018). Early fundoplication is as‐ sociated with slower decline in lung function after lung transplantation in patients with gastroesophageal reflux disease. J Thorac Cardiovasc Surg.

[BIBR-10] Fisichella P.M., Davis C.S., Gagermeier J. (2011). Laparoscopic antire‐ flux surgery for gastroesophageal reflux disease after lung transplantation. J Surg Res.

[BIBR-11] Blondeau K., Mertens V., Vanaudenaerde B.A. (2008). Gastro‐oesophageal reflux and gastric aspiration in lung transplant patients with or without chronic rejection. Eur Respir J.

[BIBR-12] Stovold R., Forrest I.A., Corris P.A. (2007). Pepsin, a biomarker of gastric as‐ piration in lung allografts: a putative association with rejection. Am J Respir Crit Care Med.

[BIBR-13] Leiva‐Juarez M.M., Benvenuto L., Costa J. (2022). Identification of lung transplant recipients with a survival benefit after fundoplication. Ann Thorac Surg.

[BIBR-14] Davidson Franklin, D Kumar (2020). Fundoplication to preserve al‐ lograft function after lung transplant: systematic review and meta‐analysis. J Thorac Cardiovasc Surg.

[BIBR-15] Latorre‐Rodríguez A.R., Razia D., Omar A. (2024). Pulmonary and esopha‐ geal function in lung transplantation: fundamental principles and clinical ap‐ plication. Transplant Rev (Orlando.

[BIBR-16] Patti M.G. (2016). Antireflux surgery in lung transplant patients. Gastroenterol Hepatol (NY.

[BIBR-17] Robertson A.G.N., Krishnan A., Ward C. (2012). Anti‐reflux surgery in lung transplant recipients: outcomes and effects on quality of life. Eur Respir J.

[BIBR-18] Eminoglu L. (2022). Presentation and outcomes of laparoscopic Nissen fundo‐ plications. Pol Przegl Chir.

[BIBR-19] Green C.L., Gulack B.C., Keshavjee S. (2023). Reflux surgery in lung trans‐ plantation: a multicenter retrospective study. Ann Thorac Surg.

[BIBR-20] Abbassi‐Ghadi N., Kumar S., Cheung B. (2013). Anti‐reflux surgery for lung transplant recipients in the presence of impedance‐detected duodenogastro‐ esophageal reflux and bronchiolitis obliterans syndrome: a study of efficacy and safety. J Heart Lung Transplant.

[BIBR-21] Yergin C.G., Herremans K.M., Patel S. (2023). Laparoscopic Toupet fundo‐ plication: a safe and effective anti‐reflux option in lung transplant recipients. Surg Endosc.

[BIBR-22] Razia D., Mittal S.K., Fournier S. (2023). Antireflux surgery versus medical management of gastro‐oesophageal reflux after lung transplantation. Eur J Cardiothorac Surg.

[BIBR-23] Razia D., Mittal S.K., Walia R. (2023). Morbidity of antireflux surgery in lung transplant and matched nontransplant cohorts is comparable. Surg Endosc.

[BIBR-24] Krahelski O., Ali I., Namgoong C. (2025). Interventional anti‐reflux man‐ agement for gastro‐oesophageal reflux disease in lung transplant recipients: a systematic review and meta‐analysis. Surg Endosc.

